# A Type 2 Diabetes Mellitus Patient With Severe Diabetic Gastroparesis Successfully Treated With Intravenous Erythromycin

**DOI:** 10.7759/cureus.49075

**Published:** 2023-11-19

**Authors:** Saori Inoue, Hiroko Yasuda, Kamiya Takashi, Hideki Okamoto

**Affiliations:** 1 Internal Medicine, Meitetsu Hospital, Nagoya, JPN

**Keywords:** erythromycin, diabetic neuropathy, diabetic gastroparesis, type 2 diabetes, diabetes mellitus

## Abstract

Gastroparesis, characterized by a decrease in gastric emptying, can lead to worsened diabetes control and a reduced quality of life. The patient was a 32-year-old male with type 2 diabetes. He was initially admitted for control of diabetes, and two months later, he was readmitted due to nausea and vomiting. He showed resistance to various drugs, including antiemetics, and an upper gastrointestinal endoscopy revealed significant gastric residue, leading to a diagnosis of gastroparesis. We administered intravenous erythromycin. After three days of treatment, the vomiting resolved. Gastroparesis is challenging to manage, and there are limited effective treatment options. We experienced a case of diabetic gastroparesis with severe vomiting and loss of appetite that responded remarkably well to intravenous erythromycin treatment. Intravenous administration of erythromycin may be a potentially effective treatment for gastroparesis.

## Introduction

Gastroparesis, characterized by delayed gastric emptying without mechanical obstruction, often presents with symptoms such as loss of appetite, nausea, and vomiting [1]. Its etiology includes idiopathic, drug-induced, post-surgical, post-infectious, and diabetic causes [2]. Diabetic gastroparesis, particularly prevalent in long-standing type 1 diabetes patients with complications [3], poses a significant clinical challenge due to its destabilizing effect on blood glucose control and its adverse impact on quality of life [4]. Treatment typically involves blood glucose management, dietary modifications, and the use of prokinetic agents, with erythromycin being considered effective, as it acts on motilin receptors to stimulate gastric emptying [5]. Clinical studies on the effects of erythromycin are limited. In a previously reported study, oral administration of erythromycin was conducted in 10 patients with diabetic gastroparesis who had received intravenous administration [6]. For both solids and fluids, it accelerated the rate of gastric emptying. Improvement in gastric obstruction symptoms was observed in one out of four patients after four weeks of administration, and two individuals who were solely on enteral nutrition resumed oral intake. We report a case in which intravenous erythromycin administration led to a remarkable improvement in symptoms in a patient with diabetic gastroparesis accompanied by frequent vomiting.

## Case presentation

A 32-year-old male with a body weight of 70 kg and body mass index of 24.8 was diagnosed with diabetes during a health check-up two years ago but had not received any treatment. He had no significant medical history but had a family history of diabetes, with his grandmother being diabetic. About a year ago, he started experiencing intermittent nausea and vomiting. He was brought to our hospital's emergency department after a week-long episode of vomiting. His glycated hemoglobin (HbA1c) was 10.9%, and his blood sugar was 299 mg/dL, indicating diabetes and ketosis. He was admitted for control of diabetes, and his nausea improved during his hospital stay. As complications of diabetes, he had peripheral neuropathy, autonomic neuropathy, proliferative diabetic retinopathy, and diabetic nephropathy: chronic kidney disease stage G1 A2 (urinary microalbumin 37.6 mg/g creatinine).

**Figure 1:**
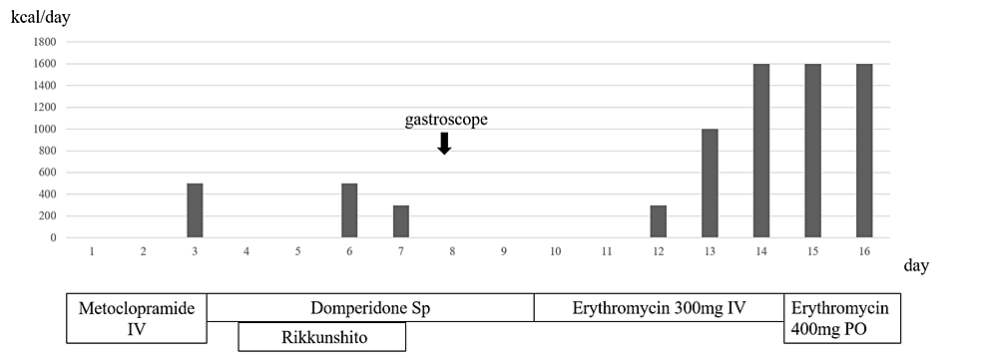
The progression of food intake and medication usage IV: intravenous injection, Sp: suppository, PO: per os

His treatment is metformin 500 mg/day, canagliflozin 100 mg/day, mitiglinide 20 mg/day, insulin degludec 8 units/day, and semaglutide 0.5 mg/week. He improved his diabetes control and continued to visit our outpatient clinic after discharge However, two months later, he was admitted due to recurrent vomiting. His blood sugar was 153 mg/dL, HbA1c was 6.4%, and mild ketosis was observed (Table 1). We administered intravenous metoclopramide, hydration, and insulin, and he commenced oral intake on the third day of hospitalization. However, he experienced vomiting again, despite the addition of domperidone suppositories and rikkunshito. He continued to have poor oral intake and persistent post-meal vomiting. Abdominal CT revealed no organic abnormalities and blood tests showed no significant abnormalities. On the eighth day of our hospitalization, he underwent an upper gastrointestinal endoscopy, which revealed significant gastric residue despite minimal oral intake the previous day, along with delayed peristalsis. We diagnosed him with diabetic gastroparesis. From the tenth day of hospitalization, intravenous erythromycin at a dose of 300 mg/day was initiated, and three days later, his vomiting improved, allowing for oral intake. On the fifteenth day of hospitalization, he was switched to oral erythromycin at a dose of 400 mg/day and was subsequently discharged (Figure 1). Two months later, erythromycin was discontinued, and there has been no recurrence of symptoms.

**Table 1:** Laboratory assessment result

Parameters	Results	Reference value
pH	7.46	7.350-7.450
HCO3-	26.7mmol/L	22-26mmol/L
Total protein	8.2g/dL	6.6-8.1g/dL
Albumin	4.7g/dL	4.1-5.1g/dL
Total bilirubin	0.68mg/dL	0.4-1.5mg/dL
Alanine transaminase	22U/L	10-42U/L
Aspartate transaminase	21U/L	13-30U/L
Lactate dehydrogenase	332U/L	4.5-18U/L
γ-glutamyl transpeptidase	13U/L	13-64U/L
Creatinine	0.58mg/dL	0.65-1.07mg/dL
Blood urea nitrogen	14mg/dL	8-20mg/dL
Creatine kinase	21U/L	41‐153U/L
Na	138mmol/L	138-145mmol/L
K	5.2mmol/L	3.6-4.8mmol/L
Cl	96mmol/L	101-108mmol/L
Ca	10.1mg/dL	8.8-10.1mg/dL
Glucose	153mg/dL	73-109mg/dL
Hemoglobin A1c	6.4％	4.9-6.0％
Total ketone body	257μmol/mL	＜130μmol/mL
Acetoacetate	119μmol/mL	＜55μmol/mL
D-3-hydroxy butylate	138μmol/mL	＜85μmol/mL
Urine-protein	+1	
Urine-sugar	ー	

## Discussion

Gastroparesis is a condition characterized by delayed gastric emptying without mechanical obstruction in the stomach, leading to symptoms such as nausea, vomiting, and abdominal fullness. It can hinder the absorption of sugars and medications, resulting in poor blood sugar control [7].

Epidemiological data on gastroparesis are limited, but the age-adjusted incidence rates per 100,000 person-years in the U.S. white population were 9.8 (95% confidence interval (CI) 7.5-12.1) for women and 2.4 (95% CI 1.2-3.8) for men. The causes included diabetes in 21.7%, connective tissue diseases in 6.0%, post-surgical gastroparesis in 3.6%, malignancy in 2.4%, psychiatric illnesses in 6%, and provocative drugs in 4.8% [8].

When gastroparesis is suspected, diagnostic procedures, such as endoscopy or barium studies, are conducted to exclude organic disorders of the gastric outlet or digestive ulcer diseases. Gastric emptying function tests are typically performed using gastric emptying scintigraphy [9], but facilities capable of conducting this test are very limited. Measurement of gastric diameter via ultrasound examination depends significantly on the operator's skill [10]. There is no established diagnostic method.

Upon admission, the patient exhibited mild ketosis, and gastrointestinal symptoms related to ketosis were considered. Despite improvement in ketosis during the hospital stay, vomiting persisted. Upper gastrointestinal endoscopy performed while fasting revealed the presence of food residue in the stomach and significant impairment of gastric emptying, leading to a diagnosis of gastroparesis. Although diabetic gastroparesis is more common in patients with a long history of diabetes, this case involved a patient with a relatively short diabetes duration of two years. However, the patient presented with advanced nephropathy, neuropathy, and retinopathy, suggesting that the duration of undiagnosed diabetes may have been longer.

There have been reports of transient worsening of neuropathy following the improvement of hyperglycemia [11]. In this case, as well, it is possible that the rapid improvement of hyperglycemia due to the hospitalization two months prior may have contributed to the progression of symptoms.

Gastroparesis treatment options include dietary modifications, lifestyle changes, medication therapy (prokinetics, antiemetics, botulinum toxin injection into the pylorus), and interventions like a nasojejunal tube or gastrostomy [12]. Medication therapy often starts with prokinetic agents that stimulate gastrointestinal motility, but their effectiveness may be limited in many cases.

Erythromycin acts on the motilin receptors to promote gastric emptying and has been reported to induce gastric contractions in both healthy individuals and patients when administered at doses of 250 mg or 300 mg [5]. In general, the administration of tablets in cases where gastric emptying is impaired is expected to be insufficient due to delayed drug absorption. Therefore, in this case, we initially chose intravenous administration of erythromycin at a dose of 300 mg/day, which can be administered non-orally, with the expectation of a reliable drug effect. Subsequently, after confirming its effectiveness, we considered switching to the tablet form of the same drug, but it was confirmed that there was no worsening of symptoms. The long-term administration of erythromycin has drawbacks due to its antibiotic nature and the development of tachyphylaxis through downregulation of motilin receptors [13]. However, in this case, we were able to discontinue the medication after two months without apparent side effects, and there have been no signs of symptom recurrence after discontinuation. Further investigation will be necessary to determine whether symptoms will recur, whether the same medication will remain effective upon recurrence, or if additional treatment options are needed.

## Conclusions

Diabetic gastroparesis is a condition for which there is no established treatment. In this case, we experienced a significant improvement in severe gastroparesis with frequent vomiting through the intravenous administration of erythromycin. There is a possibility that the intravenous administration of erythromycin may be effective for gastroparesis.
